# Cancer-related worry and risk perception in Brazilian individuals seeking genetic counseling for hereditary breast cancer

**DOI:** 10.1590/1678-4685-GMB-2019-0097

**Published:** 2020-04-22

**Authors:** Edenir Inêz Palmero, Natalia Campacci, Lavinia Schüler-Faccini, Roberto Giugliani, José Claudio Casali da Rocha, Fernando Regla Vargas, Patricia Ashton-Prolla

**Affiliations:** 1 Universidade Federal do Rio Grande do Sul, Departamento de Genética, Porto Alegre, RS, Brazil.; 2 Hospital de Câncer de Barretos, Centro de Pesquisa em Oncologia Molecular, Barretos, SP, Brazil.; 3 Hospital de Clínicas de Porto Alegre, Serviço de Genética Médica, Porto Alegre, RS, Brazil; 4 Hospital de Câncer AC Camargo, Departamento de Oncogenética, São Paulo, SP, Brazil; 5 Instituto Nacional do Câncer, Rio de Janeiro, RJ, Brazil.

**Keywords:** Hereditary breast cancer, hereditary cancer, cancer-related worry, cancer risk perception, genetic counselling

## Abstract

In Brazil, the population in general has little knowledge about genetic risks, as well as regarding the role and importance of the Cancer Genetic Counseling (CGC). The goal of this study was to evaluate cancer-related worry and cancer risk perception during CGC sessions in Brazilian women at-risk for hereditary breast cancer. This study was performed in 264 individuals seeking CGC for hereditary breast cancer. Both cancer-affected and unaffected individuals were included. As results, individuals with and without cancer reported different motivations for seeking CGC and undergoing genetic testing. A correlation was observed between age at the first CGC session and age at which the closest relative was diagnosed with cancer. Multivariate analysis showed that educational level, cancer risk discussion within the family, and number of deaths by cancer among first-degree relatives influenced positively the cancer risk perception. In conclusion, the results of this study indicate that cancer-related worry and cancer risk perception are significant aspects of morbidity in individuals seeking CGC, whether they are cancer-affected or unaffected. CGC has an important role in health education and cancer prevention for its potential of promoting an accurate perception of the risk.

## Introduction

The development and rapid improvement in molecular diagnosis of inherited genetic diseases during the last decades resulted in an increasing need for genetic counselling (GC), since it is a process that provides information and support for at-risk individuals and their families. This is also applicable to individuals at-risk for a specific group of genetic disorders, the hereditary cancer syndromes. As cancer is usually diagnosed during adult life and can be prevented or diagnosed in very early stages, GC may be particularly important, since predictive testing of at-risk individuals may guide effective cancer prevention strategies (Heiniger *et al*., 2015). Several reports indicate that well informed patients understand better their risk of developing cancer and this, in turn, facilitates the complicated decision making process regarding screening and prevention options for these high-risk individuals (Stopfer 2000; Hampel 2002).

Cancer Genetic Counselling (CGC) services have been established in many countries. They have developed quickly, mainly from the beginning of the decade of 1990, when several high-penetrance cancer predisposition genes, such as *BRCA1* and *BRCA2* were identified (Epplein *et al*., 2005). In Brazil, a number of public CGC services have been created over the last decade. They are predominantly located in tertiary university hospitals of several Brazilian capitals and offer genetic counselling through the public health care system for the index patient and at-risk family members and are directed to a selected population of highrisk patients that are seen in oncology clinics of these institutions (Penchaszadeh 2001; Llerena Junior 2002; Palmero *et al*., 2016). Four of these public CGC services, established in Porto Alegre (RS), São Paulo (SP), Barretos (SP) and Rio de Janeiro (RJ), are currently considered reference centers for cancer research and/or GC in the country. Genetic counselling for hereditary breast cancer in these services is performed, including medical and family histories with construction and analysis of pedigrees, determination of cancer risk estimates and of the prior probability of mutations in cancer predisposition genes such as *BRCA1/2.* Genetic testing is offered to those families that fullfill NCCN *(National Comprehensive Cancer Network)* criteria for a hereditary breast cancer syndrome through local, national and/or international collaborative research studies (Palmero *et al*., 2007), once genetic testing is not covered by Brazilian Public Health System.

During CGC, the individual cancer risk perception, defined as the “subjective estimation of the likelihood that one might be diagnosed with cancer in the future”(Honda *et al*., 2004), is usually assessed and discussed. It is known that individuals exposed to similar risk factors can perceive them differently, depending on individual personality traits, life style, number of relatives affected by cancer and their age at diagnosis, and susceptibility to the influence of mass media (Hampel 2002; Heiniger *et al*., 2015). Significant attention has been given to the study of cancer risk perception, for its supposed influence on adherence to screening and preventive interventions (Leventhal *et al*., 1999; Eismann *et al*., 2016). In addition, some authors have demonstrated that perceived risk has a stronger impact on cancer preventive intentions than objective risk (van Dijk *et al*., 2003).

Hardly any data exist on cancer-related worry and cancer risk perception in South American patients seeking GC for hereditary breast cancer (HBC) risk. This is a particularly interesting population because genetic testing for cancer predisposition is not easily and readily available to most people that rely upon the public health care system and the population, sometimes, do not have enough knowledge concerning hereditary cancer (Campacci *et al*., 2015). Besides, Brazil has a highly heterogeneous population, with enormous diversity of health culture, religiosity and economic status; which may affect the understanding of the GC process. The present study was conducted to characterize these aspects in a group of individuals seeking CGC in Brazilian reference centers, with identification of different factors that may influence these parameters. Both patients with and without a previous history of cancer and patients at different levels of risk for HBC were studied.

## Material and Methods

### Study design and patient recruitment

This study included 264 individuals seeking GC for hereditary breast cancer in CGC services of the Brazilian public healthcare system during a period of 15 months. Patients were all older than age 18 years and were recruited in three capitals after signature of informed consent: Porto Alegre (RS), São Paulo (SP) and Rio de Janeiro (RJ). Ethical approval was obtained from the respective institutional ethics and research committees. At inclusion, informed consent was obtained from all individual participants included in the study. Participants were assigned to two groups: (1) cancer-affected individuals with a family history suggestive of HBC, or (2) asymptomatic individuals with a family history suggestive of an HBC syndrome. All participants were asked to complete a cancer-related worry and perceived risk questionnaire before the first GC consultation, and had their pedigree and previous/present medical history recorded. Breast cancer screening procedures (adherence and periodicity) were recorded for all women. Using standard breast cancer screening guidelines (Brandt *et al*., 2002) and age, women were classified regarding adherence to and periodicity of each screening procedure as normovigilant (adopting recommended guidelines), hypovigilant (performing less than the recommended procedures for age) or hypervigilant (performing screening procedures more often than recommended by standard guidelines). Mutation prevalence tables were used to estimate prior probability of a *BRCA* mutation in all families (Frank *et al*., 1998).

### Study measures

The following sociodemographic variables were recorded: sex, age, marital status, educational level, and number of children. Cancer risk perception and cancer-related worry were assessed through specific tools (sections 2.3 and 2.4). Additional information obtained included: patients’ understanding about breast cancer etiology, knowledge about genetic testing for HBC, habit of discussing cancer-related issues with relatives, presence of a relative during CGC sessions and motivation for GC and genetic testing. Detailed information on personal breast (and other) cancer history, breast cancer screening (performance and periodicity), and family history of cancer were also recorded.

### Cancer risk perception

Perceived lifetime risk of cancer (for self and other relatives) was assessed by four questions adapted from (Lerman *et al*., 1994,^1995^; van Dijk *et al*., 2003) as follows:

- Numerical lifetime risk of developing cancer was measured with the question “How would you rate your lifetime risk of developing cancer” [or “(...) developing cancer again”] for the affected individuals and scored by a visual analogic scale rating risk from 0-10 (expressed in %).
- Verbal risk of developing cancer was measured by the question “What do you feel is your lifetime risk of developing cancer” [or “(...) developing cancer again”] for the affected individuals and answers were scored from 1 to 4 (low, moderate, high, very high).
- Relative lifetime risk of developing cancer was measured with two questions: “What do you feel is your lifetime risk of developing cancer compared to other persons of your age” and “How would you rate the lifetime risk of your close relatives developing cancer” [or “(...) developing cancer again” ] for the affected individuals. Answers to the first question were scored from 1 to 5 (null, lower, equal, higher, much higher). Answers to the second question were evaluated by a visual analogic scale rating risk from 0-10 (expressed in %). Since this is a descriptive study, results are preliminary and no validation was performed at the time of recruitment or before. The instruments used were posteriorly valeted for the Portuguese language with no major modifications in relation to the original version in English.

### Cancer-related worry

Cancer-related worry and its impact on daily life were assessed by the same instrument using two questions adapted from (Lerman *et al*., 1994,1995): “How often do you worry about the possibility of having cancer (or developing cancer again)” and “Does your cancer-related worry interfere with your daily activities”. Both questions used a visual analogic scale and answers were scored from 0 (never) through 5 (about half of the time) to 10 (all the time). Since this is a descriptive study, results are preliminary and no validation was performed at the time of recruitment or before. The instruments used were posteriorly valeted for the Portuguese language with no major modifications in relation to the original version in English.

### Statistical analysis

SPSS version 19.0 was used for data handling and statistical analyses. For descriptive analysis, categorical variables were described by their absolute and/or relative frequencies and quantitative variables were expressed as mean ± SD or median and range (i.e. Table 1, Sociodemographic characteristics, cancer risk estimates and prior probabilities of mutation in a *BRCA* gene). For analytical statistics, Student’s t-test for independent samples or ANOVA for more than two groups were used to compare symmetric variables. Mann-Whitney and Kruskal-Wallis tests were used when comparing asymmetric variables in two or more groups, respectively (non-parametric tests were used when appropriate due to skewed distributions or small sample size). Categorical variables were examined by Chi-square and multiple comparisons or Fisher’s exact test and the correlation coefficient of Spearman was used whenever analysis included two quantitative variables. Crohnbachs coefficient was used to verify internal consistency among questions of the instrument used to measure risk perception. In addition, multivariate analysis was performed to identify variables that may influence cancer risk perception and cancer-related worry. The variables included in the analysis were: previous diagnosis of cancer, educational level, discussion within the family about cancer-risk, presence of relatives during the GC session, number of deaths by cancer among first-degree relatives and perceived risk of cancer for other relatives.

**Table 1:** Sociodemographic characteristics, cancer risk estimates and prior probabilities of mutation in a *BRCA* gene for the 264 individuals included in the study (total and according to cancer status).

	N (%)	Affected N (%)	Unaffected N (%)	*p* value
**Sociodemographic variables**				
**Gender**				
Female	253 (95.8)	146 (55.3)	118(44.7)	>0.05
**Age (years)**				
Mean ± SD	45.7 + 11.8	49.30 + 11.2	41.40 + 11.2	<0.001
**Marital status**				
Married or cohabitating	163 (61.7)	97 (66.4)	66 (55.9)	0.105
**Children**				
Yes	195 (73.9)	123 (84.2)	72 (61.0)	< 0.001
**Educational Level**				
Elementary	38 (14.4)	33 (22.6)	5 (4.2)	< 0.001
High school	36(13.5)	25 (17.1)	11 (9.3)	
College/University	190 (72.1)	88 (60.3)	102 (86.4)	
**Prior probability of mutation in** *BRCA* gene (%)				
Median	13.3 (7.6-18.0)	18.0 (7.6-18.8)	7.6 (4.1-20.5)	0.105
Cancer risk perception (lifetime)*				
Median		40.0 (10.0-50.0)	50.0 (40.0-60.0)	0.003
**Cancer-related worry**				
Median	-	50.0 (20.0-100.0)	50.0 (30.0-80.0)	0.866

Numerical perceived lifetime risk of developing cancer (assessed by a visual analogic scale). For patients affected with cancer, a question was made regarding development of a new tumor.

## Results

The sociodemographic characteristics of the 264 individuals included in the study are summarized in Table 1.

The proportion of cancer-affected and unaffected individuals was similar. However, cancer-unaffected individuals were younger and reported higher educational levels *(p* < 0.01) (Table 1).

**Figure 1:**
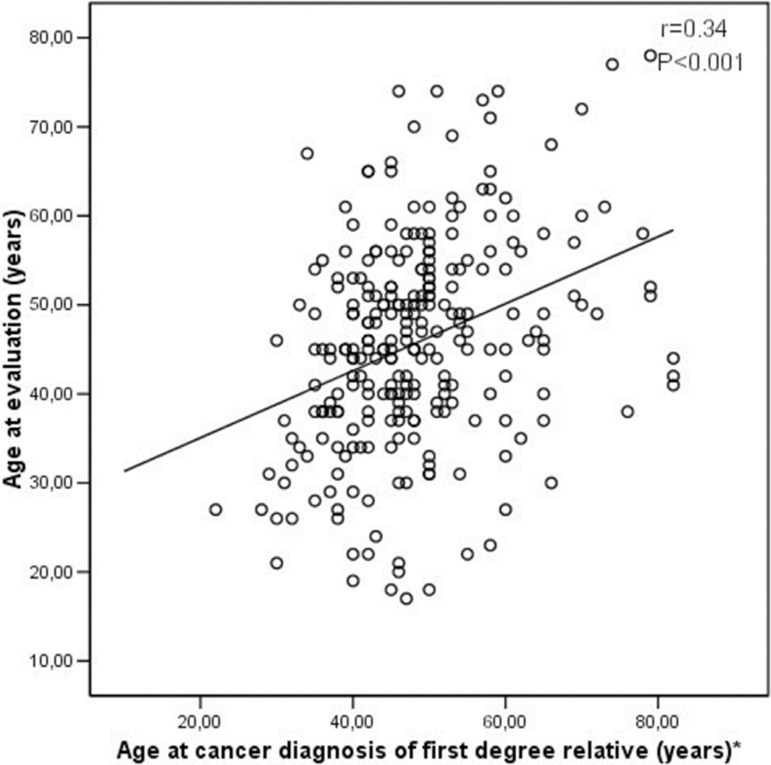
Correlation between individuals age at first CGC session and age of cancer diagnosis in closest relative.

Among cancer-affected patients, the main reasons for seeking CGC were: referral by their primary care physician, concern with cancer history in the family, desire to prevent the occurrence of additional cases in the family and previous personal cancer history. Most of the asymptomatic individuals indicated that the reasons for seeking such evaluation were: (a) they had reached the age at which a close relative had been diagnosed with cancer (33.0%) and (b) they had a high number (3 or more) of cancer diagnoses in the family (31.5%). A correlation *(p* < 0.001) was observed between age at the first CGC session and age at which the closest relative was diagnosed with cancer (Figure 1). A significant number of individuals (N=135; 51.1%) were accompanied by a relative during the CGC process and reported frequent discussions of cancer-related issues with their relatives (N=172; 65.1%). Relatives were more often present in CGC sessions of patients who reported usual discussions about cancer-related issues within the family (p=0.015).

The proportion of women reporting regular breast self-examination, mammography screening and breast examination by a health care professional was 52.2%, 92.0%, and 76.3%, respectively (data available for 253 women). In the group of women older than 40 years (information available for 147 women), the majority (n=90; 61.2%) were classified as hypovigilant, 15.7% (n=23) were classified as normovigilant, and 23.1% (n=34) as hypervigilant. No correlation was observed between educational level and adherence to screening procedures (p=0.269). The occurrence of familial cases of cancer was ascribed to “genetic factors” by 45.6% of the patients, common exposure to environmental factors by 22.6% and other factors (i.e. “Gods will” or “Bad luck”) by 31.7%.

Patients showed some familiarity with the existence of cancer predisposition testing: 74.8% mentioned they had heard about such test. Educational level and knowledge about the availability of genetic testing were significantly correlated *(p* < 0.001). Reasons for undergoing genetic testing (in case HBC criteria were met) were evaluated for patients with and without cancer and results are presented in Table 2.

**Table 2:** Reasons for undergoing genetic testing as reported by cancer-affected and unaffected individuals.

Reasons	Affected N (%)	Unaffected N (%)	*P*
To contribute with science and research	19(13.0)	10(8.5)	0.323
To understand the cause for the multiple cancer cases in my family	13 (8.9)	14(11.9)	0.541
To know as soon as possible my risk of developing cancer and decide what to do to decrease this risk	30 (20.5)	81 (68.6)	< 0.001
For my children and other relatives	79 (54.1)	13 (11.0)	< 0.001
To follow the advice of doctors if they felt I should be tested	5(34)	0 (0)	0.070

The analysis of pedigrees revealed that 87.1% of the probands had at least one deceased relative affected with cancer and 50.4% reported at least one first-degree relative affected by and deceased with cancer.

Prior probability of carrying a mutation in a *BRCA* gene was estimated with mutation prevalence tables *(BRCA1* and *BRCA2* Prevalence Tables by Myriad Genetics) (Table 1).

Perceived numerical lifetime risk of developing cancer (assessed by a visual analogic scale) was in average 44.5% with a median of 50.0%. It was perceived as < 10% by 21.2% of the patients, between 11-29% by 8.0%, and 330% by 70.8% of the individuals. Perceived verbal risk of developing cancer (classified as low, moderate, high or very high) was described as very high or high by 64.0%, moderate by 24.6% and low by 11.4% of the individuals.

Perceived relative lifetime risk of developing cancer in comparison to other persons of the same age was described as higher or much higher by 68.6% of the individuals. Relative lifetime risk of developing cancer in comparison to other family members (assessed by a visual analogic scale) was in average 45.5% with a median of 50.0%. It was perceived as < 10% by 19.3% of the patients, between 11-29% by 7.6%, and 3 30% by 73.1% of the individuals. A statistically significant correlation was observed between perceived lifetime risks of cancer for self and other relatives *(p* < 0.001). Furthermore, internal consistencies of answers to all four questions related to perceived risk of cancer was determined and found to be acceptable (Cronbachs µ 0.856).

Regarding cancer-related worry, all individuals included in the study indicated that they have this concern at least sometimes, 47.0% worry half of the time or more and 24.2% worry all the time. When asked about the frequency with which this worry interferes with daily activities, most individuals (56.4%) indicated that interference occurred in 20.0% or less of the time. However, when analysing only those individuals that worry all the time about cancer, in approximately one third of those (34.4%) worry interfered in daily activities more than 80% of the time. Statistically significant correlations were observed between perceived risk of cancer and both, cancer-related worry and frequency of interference of this worry with daily activities *(p* < 0.001). Comparisons of perceived numerical lifetime risk of developing cancer and cancer-related worry were performed for cancer-affected and unaffected individuals and are summarized in Table 2.

Perceived chance of cure of cancer in general was considered very high by 6.8% (n=18) of the patients, high by 29.2% (n=77), and low by 37.5% (n=99) of the patients. Sixty-three patients (23.9%) considered cancer an incurable disease, and 7 (2.6%) did not answer. There was a poorly significant negative correlation between perceived risk of cancer and perceived chance of cure (r=-0.19; *p*=0.002). Interestingly, there was no correlation between perceived chance of cure and the number of patients affected with and the number of deaths by cancer (p=0.547) in the family. However, when only first-degree relatives were considered in the analysis a trend towards a positive correlation was observed (p=0.134).

Finally, multivariate analysis was performed to identify variables which could influence risk perception. A statistically significant correlation was observed between risk perception and the following: educational level, risk discussion within the family, and number of deaths by cancer among first-degree relatives.

## Discussion

Only few studies have been conducted with South American patients about the role and impact of CGC on cancer-affected patients and their relatives. Little is known about these individuals cancer-related worry and risk perception and even less is known about the influence of these on the CGC process. The present study describes a sample of patients seeking GC for hereditary breast cancer in Brazilian public CGC reference centers, regarding several aspects of their medical and family histories, cancer-related worry and. cancer risk perception.

The sample itself was distributed over a broad range of ages (18 and 78 years) composed mainly by women, and more educated than the average patient in the public Brazilian health care system (DATASUS 2006). Cancer-unaffected individuals in this sample were younger and had an average higher educational level than cancer-affected patients which is likely a reflection of a higher interest in understanding the causes of disease and in cancer prevention in the former group.

It is noticeable that a significant number of individuals were accompanied by relatives not only in their first consultation but during the entire GC process, supporting the concept that cancer has an impact upon the entire family (MacDonald *et al*., 2010) and that GC must be directed not only to the index case but also to other at-risk individuals (Douglas *et al*., 2009). In addition, most patients in our study indicated that cancer occurrence and recurrence were discussed within the family, and perceived the risk of developing cancer for self and for their relatives to be high.

A strong influence of family history on these individuals was observed when the reasons for seeking GC were recorded, especially for cancer-unaffected individuals. For one third of these, the main reason for seeking CGC was the proximity to the age at which a close relative was diagnosed with cancer; in another third, the presence of a high number of cancer-affected relatives was reported as the main reason. These results are in accordance with previous findings that suggested that “the fear to develop cancer increases with the proximity to the age at which a close relative developed the disease” and that “women’s perceptions of vulnerability may reflect this `lived experience’ of cancer (in the family), through strong identification with an affected or deceased mother or sister” (Kash *et al*., 1995; Hopwood 2000). In a similar study of the motivation for CGC among cancer affected and unaffected individuals, in the former, understanding of personal risk for developing cancer was the main drive to seek CGC (Huiart *et al*., 2002).

When asked about the single most important reason for undergoing genetic testing, most cancer-affected patients stated that it would be to help their children or other relatives. Cancer-unaffected individuals were mainly concerned with a more objective risk determination and indicated that they would decide to undergo testing to know as soon as possible their own risk of developing cancer and their options for cancer prevention. According to Brandt *et al*. (2002) an unaffected woman may be more concerned about her own risk and may feel that, since she is unaffected, her childrens risk are to some degree a lower priority (Brandt *et al*., 2002). Women who have had the disease may worry more about their children with respect to cancer risk because the risk is “more real and more direct”. Thus, an important conclusion that can be drawn from these observations is that, cancer-affected and unaffected individuals that seek cancer genetic counselling, even if members of the same family, may have different needs and expectations.

Although 45.6% of the patients associated the occurrence of several cancer cases in their families to possible genetic risk factors, a significant proportion of individuals attributed this history to “bad luck” and “Gods will”. Finally, 24.6% of the patients were unaware of the possibility of genetic testing for cancer predisposition. As expected, unawareness was higher in individuals with less education.

The perceived lifetime risk of cancer was significantly high for the vast majority of individuals. These results were similar to those observed in other studies. Among 503 women at-risk for hereditary breast and ovarian cancer seeking CGC in the U.S., 80% overestimated their risk for breast cancer by up to 4 fold (Kash *et al*., 1995). Other studies also reported this increased perception. An important consequence of this exaggeration in perceived risk, which was reported in the above mentioned studies and was also observed in our sample, is the poor adherence to cancer screening programs (Evans *et al*., 1993; Ritvo *et al*., 2002; McAllister 2003; Watson *et al*., 2004). Although implicated in some studies, an influence of lower educational levels on adherence to breast cancer screening recommendations was not observed in our study.

An exaggeration of perceived risk may be in itself harmful, inducing people to misconceptions about the causes, possible treatment options and recurrence of cancer. Usually, high cancer risk perceptions are associated with higher levels on cancer-related worry, and this also occurred in our study. As shown by some authors, absolute and comparative risk perceptions may be independent predictors of cancer worry and may have a different impact depending on the individuals gender, personal level of psychological distress and cancer status (affected versus unaffected) and therefore knowledge of these variables during the process of CGC is important to determine which risk communication strategy is more effective in a given situation (Zajac *et al*., 2006). Furthermore, increased levels of anxiety related to an unreal perception of risk may lead to treatment abandonment or, in the extreme, unnecessary interventions. Proper CGC intends to determine the individuals lifetime risks of developing cancer and the prior probability of mutation in a predisposition gene based on family histories. In many cases these estimates may be refined and confirmed in a more objective way through genetic testing. When effective, these strategies have shown to increase understanding and acceptance of risk as well as improve adherence to adequate screening and /or prevention guidelines. An accurate risk perception allows for an increase in quality of life for patients with high levels of anxiety and stress related to an exaggerated perception of breast cancer risk, and reduces excessive and unnecessary examinations and screening procedures (Bottorff *et al*., 2004).

Work published by d’Agincourt-Canning (2005) explores the effect of experiential knowledge on construction of risk perception and suggests that knowledge derived from experience often takes precedence over objective clinical estimates of risk. Therefore, the author further suggests that a CGC strategy that explores the patients lived experience and knowledge of cancer may enhance communication of genetic risk and help modify exaggerated and/or inaccurate risk perceptions. This maybe a particularly important approach in a population such as ours were the counselee seems to have such a close relationship to other family members.

Finally, the way of dealing with risk is not only influenced by the individuals perception but also by his/her culture. Hofstede (1997) studied different cultures from nations around the world using five parameters, one of them being the uncertainty avoidance index (UAI). This index reflects the tolerability of a certain society towards uncertainty and ambiguity. Groups that have a high UAI have low tolerance to these features and create strategies to control or avoid risk and ambiguity. The Brazilian society has a higher UAI as compared to countries like Denmark, the United Kingdom and the United States of America, but lower than those of France, Spain and Portugal. These cultural aspects may also interfere with cancer risk perception and cancer-related worry in a CGC setting involving individuals from this cultural background, and further studies should be undertaken to address these.

In Brazil, the population in general has little knowledge about their genetic risks, as well as available opportunities of risk reduction through preventive measures. In addition, cultural aspects indicate that it is a society that has a high propensity to avoid risk and uncertainty results of this study indicate that cancer risk perception and cancer-related worry are a significant aspect of morbidity in individuals seeking CGC, whether they are cancer-affected or unaffected, and reinforce that the counselor must be aware of the influence of these factors upon the patient, his/her family and the counseling process itself. CGC may have an important role in health education and cancer prevention for its potential of promoting a more accurate perception of the risk. Genetic counseling may be instrumental to reinforce routine guidelines of breast cancer prevention, such as periodic mammographic examinations in women of different risk categories. For women such as those studied here, most of whom did not comply as expected for their age with screening guidelines, this aspect of counseling could be particularly important.

The periodic analysis of the motivation, clinical features and risk profile of patients that are evaluated in cancer genetic clinics, as well as identification and understanding of the barriers to reach such individuals is essential to enable proper communication strategies in order to effectively achieve cancer risk reduction in genetically predisposed individuals.

In this study was possible to indicate that cancerrelated worry and cancer risk perception are significant aspects of morbidity in individuals seeking CGC, whether they are cancer-affected or unaffected, and should always be addressed during counseling.

It is important to considering the CGC setting including the culture and socioeconomics aspects to execute a coherent CGC, since this process has an important role in health education and cancer prevention for its potential of promoting a more accurate perception of the risk.
